# Type 2 Transglutaminase in Coeliac Disease: A Key Player in Pathogenesis, Diagnosis and Therapy

**DOI:** 10.3390/ijms23147513

**Published:** 2022-07-06

**Authors:** Gaetana Paolella, Silvia Sposito, Antonio Massimiliano Romanelli, Ivana Caputo

**Affiliations:** 1 Department of Chemistry and Biology, University of Salerno, 84084 Fisciano, SA, Italy; ssposito@unisa.it (S.S.); aromanelli@unisa.it (A.M.R.); 2 European Laboratory for the Investigation of Food-Induced Diseases (ELFID), University of Salerno, 84084 Fisciano, SA, Italy

**Keywords:** transglutaminases, type 2 transglutaminase, coeliac disease, gluten, autoimmunity, anti-TG2 antibodies, TG2 inhibitors

## Abstract

Type 2 transglutaminase (TG2) is the main autoantigen in coeliac disease (CD), a widespread inflammatory enteropathy caused by the ingestion of gluten-containing cereals in genetically predisposed individuals. As a consequence, serum antibodies to TG2 represent a very useful marker in CD diagnosis. However, TG2 is also an important player in CD pathogenesis, for its ability to deamidate some Gln residues of gluten peptides, which become more immunogenic in CD intestinal mucosa. Given the importance of TG2 enzymatic activities in CD, several studies have sought to discover specific and potent inhibitors that could be employed in new therapeutical approaches for CD, as alternatives to a lifelong gluten-free diet. In this review, we summarise all the aspects regarding TG2 involvement in CD, including its enzymatic reactions in pathogenesis, the role of anti-TG2 antibodies in disease management, and the exploration of recent strategies to reduce deamidation or to use transamidation to detoxify gluten.

## 1. Introduction

Transglutaminases (TGs) form a broad family of enzymes with members throughout the animal world, as well as in bacteria and in plants. They mainly catalyse post-translational modifications of proteins, producing covalent cross-links with stabilising functions [1]. For example, Factor XIIIa produces the fibrin clot in the final stage of blood coagulation, prostate (type 4) TG has a role in coagulating the mammalian semen, epidermal (type 3) TG (TG3) stabilises the skin cornified envelope, and microbial TG (TGm) is employed as cross-linking agent in food and bioplastic industries. Among TGs, type 2 TG (TG2) has become famous because a simple antigenic test searching for serum anti-TG2 antibodies can confirm or exclude a diagnosis of coeliac disease (CD), an inflammatory autoimmune condition common throughout the world [2]. Regarding CD, TG2 is not only the main autoantigen; it represents one of the most important pathogenetic agents in the disease, and as a consequence, it is one of the possible targets of studies aiming to develop new therapeutical strategies [3]. Moreover, TGs, and in particular TGm, are also suitable as biotechnological tools to modify gluten proteins, which are the main environmental triggers for CD [4]. In this review, we explored all aspects regarding TGs—in particular TG2—in CD, including the consolidated concept of the role of TG2 in disease pathogenesis, the new studies on the coeliac cellular phenotype, the recent discoveries with regard to targeting TG2 as a possible new therapeutic approach, and finally, the use of a food-grade TGm to detoxify gluten sequences.

## 2. Coeliac Disease (CD)

CD is a multifactiorial enteropathy that affects the small intestine of genetically predisposed individuals [5]. A condition of partial to total atrophy, together with crypt hyperplasia and consistent lymphocytic infiltration, characterises the intestinal mucosa of affected patients. The main environmental trigger is a heterogenic proteic component of some dietary cereals, commonly known as gluten. A strong immune response against gluten, both cellular and humoral, is mounted in CD, accompanied by a humoral autoimmune response against self-proteins, in particular TG2.

### 2.1. Epidemiology and Clinical Manifestations of CD

CD is widely distributed throughout the world, affecting on average 1% of the population, with differences in incidence due mostly to the dietary habits of certain countries, or to geographical area [6]. This enteropathy can manifest at every stage of life, with a higher frequency in children between 1 and 2 years of age. Classical presentation of CD includes gastrointestinal symptoms, such as malabsorption, chronic diarrhoea, abdominal distension, and pain. In paediatric populations, the disease also presents with failure to thrive and delayed puberty. Other frequent symptoms, mainly due to nutritional deficiencies resulting from the mucosal lesions, are anaemia, osteoporosis, and weight loss [6]. Furthermore, extraintestinal manifestations are also commonly associated with CD, such as liver, skin, endocrine, and neurological disorders [7].

### 2.2. The Main Environmental Trigger in CD: Gluten

Common cereal seeds possess abundant storage proteins, 75–80% of which are gluten, a complex mixture of proteins that, due to their high content of Pro and Gln residues in their primary structure, are defined as prolamins. Gluten prolamins of wheat were the first to be identified, and are usually divided into two classes on the basis of their solubility: gliadins (the alcohol-soluble fraction, monomeric) and glutenins (the water-soluble fraction, polymeric) [8]. The equivalents of gliadin proteins in other cereals assume different names, depending on the specific cereal: secalins in rye, hordeins in barley, and avenins in oat. Polypeptide sequences responsible for the immune response in CD individuals are mainly from gliadin and related prolamins [9]. Gliadin comprises three major groups of proteins, namely *α*/*β*, *γ*, and *ω*, according to their electrophoretic mobility (*α*- and *β*-gliadins migrate separately but are very similar) [8]. *α*-gliadins contain the most immunogenic sequences characterised in the context of CD [10]. In particular, the central domain contains the 33-mer segment, comprising six overlapping epitopes relevant for CD pathogenesis. In addition, the high Pro residues content (about 15%) renders gliadin quite resistant to digestive enzymes; as a consequence, numerous large undigested peptides may come into contact with the intestinal barrier, triggering pathways leading to inflammation and immune response [11]. The immune response requires that gliadin peptides are specifically recognised by antigen-presenting cells (APCs) and presented to competent T cells. Immunogenic properties of gliadin sequences are also related to a high content of Gln residues (about 30% of total residues). Some of these Gln residues have a key role in the interaction with the cellular proteins necessary for antigen presentation to the immune system, and may be the target of a post-translational modification (i.e., TG2 deamidation) that renders them more immunogenic [2,5,12].

Even if gluten is the main environmental stimulus in the onset of CD, recent evidence has demonstrated that the alteration in the gut microbiota (a decrease in Bifidobacteria and an increase in Bacteroides has generally been observed) may represent a key environmental factor contributing to the disease [13]. Moreover, the relative timing of gluten introduction into the infant diet and recurrent gastrointestinal viral infections, in particular rotavirus infections, seem to be important in altering the intestinal barrier, thus favouring exposure to gluten [6,12,14].

### 2.3. The Genetics of CD

The main genes involved in predisposition to CD are particular haplotypes of the human leukocytes antigen (HLA) system of class II [5]. They codify for integral membrane heterodimers, which have the function of binding gluten peptides on the cell surface and presenting them to CD4^+^ T lymphocytes. Haplotypes DQ2 and DQ8 are present on APCs in 95% and 5% of CD patients, respectively [15]. However, more than 40% of individuals in the general population present these same haplotypes [16]; thus, it is clear that their presence is a necessary condition but not sufficient on its own to cause CD. Many other non-HLA genes seem to be associated with CD; large scale genomic studies identified several genetic regions harbouring genes controlling immune functions, but altogether they account for only about 15% of the genetic component of the disease [17]. Considering all HLA and non-HLA genes, it is possible to explain about 50% of CD heritability; the remaining part of the heritability could be due to the presence of rare genetic variants with a very low allele frequency, normally excluded from genome-wide association [17]. A recent and promising area of research is the exploration of CD susceptibility beyond genetics: emerging evidences have highlighted a role in CD pathogenesis of specific patterns of DNA methylation and chromatin accessibility, as well as of peculiar transcription patterns of non-coding RNA, specifically micro-RNA and long-noncoding RNA [17].

### 2.4. The Adaptive and Innate Immune Response in CD

**Figure 1:**
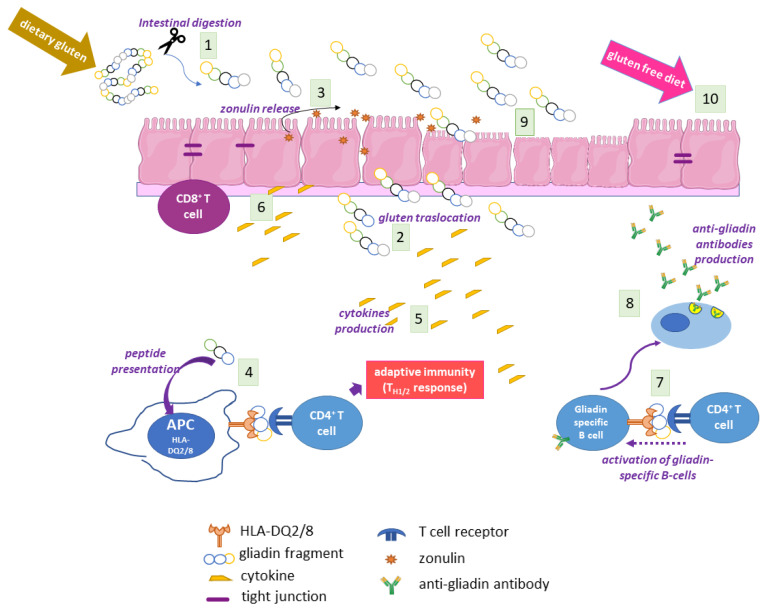
Scheme of the main events of CD pathogenesis. In gut lumen, gluten/gliadin is partially digested (**1**) and large fragments cross the intestinal barrier (**2**), triggering an increased intestinal permeability with zonulin release (**3**). Antigen presentation by HLA-DQ2/8 APCs (**4**) elicits an adaptive immune response with the release of pro-inflammatory cytokines (**5**), the recruitment of cytotoxic T cells (**6**), and the activation of gliadin-specific B cells (**7**), with the consequent production of anti-gliadin antibodies (**8**). All together, these events lead to the loss of mucosal integrity (**9**), which is slowly recovered with a gluten-free diet (**10**). This scheme does not show the role of TG2 gluten modifications, which are explained in the next figures.

Large partially digested gluten peptides in the gut lumen of CD subjects trigger a cascade of events that ultimately cause mucosal lesions, involving both the adaptive and the innate immune system [12] (Figure 1). One of the first events is gluten-induced zonulin release by intestinal epithelium, which causes an increased permeability, thus favouring gluten translocation to lamina propria [18]. Here, some gliadin peptides are modified by TG2 at the level of specific Gln residues, thus becoming more immunogenic. Both unmodified and TG2-modified gliadin peptides are presented by HLA-DQ2/8 APCs, eliciting T helper cell recruitment and activation, with the release of a cascade of pro-inflammatory cytokines, mainly interferon *γ* (INF*γ*) and tumour necrosis factor *α* (TNF*α*), and activation of B cells secerning anti-gliadin or anti-TG2 antibodies. T_H1_ and T_H2_ adaptive responses to gluten are accompanied by an innate immune response with secretion of interleukin (IL)-15, IL8, and keratinocyte growth factor, and with expansion of CD8^+^ cells; all these events, together with an increased matrix remodelling by metalloproteinases, cause extensive mucosal damage characterised by hyperproliferation, cell death and loss of epithelial barrier architecture [5,6,12]. The innate component of the immune response is strictly related to the presence of specific non-immunogenic peptides, such as the 25-mer, i.e., the sequence 31–55 of *α*-gliadin and shorter peptide 31–43 (P31–43) and peptide 31–49, which reach lamina propria, where they may induce stress signalling, proliferation, cytoskeleton alterations, and vesicular trafficking modifications [19].

### 2.5. Autoimmunity in CD

In the serum and intestinal mucosa of CD patients, antibodies to self-components can be detected; thus, CD displays the characteristics of an autoimmune disease. Some decades ago, Dieterich and colleagues demonstrated that TG2 was the main autoantigen in CD [20]. During CD onset, anti-TG2 antibodies of IgA class appear at a very early stage in the CD intestinal mucosa; in this location, they are detectable before the mucosal changes typical of CD lesions, and before they can be found in serum [21]. Then, they cross the blood vessels and reach various tissues and organs, where they accumulate [22]. Autoantibodies directed against other TG isoforms (TG3 and neuronal TG) have also been found, associated in particular with the occurrence of extra-intestinal manifestations of CD [23,24].

### 2.6. The Coeliac Cellular Phenotype

Several recent studies have highlighted that in CD cells, it is possible to observe some constitutive features that are independent from the exposure to gluten, and are also visible far from the intestine, i.e., the main site of inflammation during the response to gluten. The so-called “coeliac cellular phenotype” is an ensemble of characteristics displayed by the CD cells of duodenal biopsies or other cells, such as skin-derived fibroblasts, and can be partially reproduced in cells from control subjects when exposed to *α*-gliadin P31–43; moreover, this phenotype also includes the fact that some biological effects of P31–43 become evident in CD cells but not in non-CD cells [25,26,27]. It has been demonstrated that CD skin-derived fibroblasts have a higher content of phosphorylated proteins and a higher number of focal adhesions than cells from control subjects [25]. Furthermore, CD enterocytes and dermal fibroblasts show a delay in the progression of vesicular early endosome to late endosome, associated with increased signalling, actin rearrangement, and augmented proliferation [26]. Structural cytoskeleton alterations, associated with an increased Rho A activity, have also been described in the dendritic cells of CD children [28]. Finally, in CD enterocytes, several inflammation markers have been found to become constitutively enhanced [29]. Thus, it is reasonable to assume that CD cells possess constitutive altered pathways and responses that render them more sensible to the effects of gluten peptides and other proinflammatory agents.

### 2.7. CD Diagnosis

Currently, a single direct approach to the diagnosis of CD is not available. Genetic tests aimed at finding HLA-DQ2/8 haplotypes have only a negative predictive value, and are employed to support diagnosis. The gold standard for CD diagnosis is obtained by combining the analysis of the duodenal biopsy with the detection of serological markers [30]. Morphological evaluation of biopsy samples highlights the presence of typical features of CD mucosal lesions, i.e., flat and hyperplastic mucosa at various degrees together with an increased concentration of intraepithelial lymphocytes [5]. Currently, two types of non-invasive serological markers can be used to support a correct diagnosis: antibodies raised against the environmental trigger, in particular gliadin, and antibodies against self-components, mainly anti-TG2 antibodies. Moreover, the discovery of CD-associated circulating miRNA associated with CD could open the way for the development of new potential non-invasive biomarkers of CD [31].

#### Enzymatic and Immunofluorescence Assays

The detection of specific antibodies is routinely achieved by enzyme-linked immunosorbent assays (ELISAs) or by indirect immunofluorescence assays (IFAs). Antibodies related to CD are of both the IgA and IgG classes; however, the highest sensitivity and specificity is reached by tests detecting IgA [32]. Anti-gliadin antibodies were the first serological marker detected and quantified by ELISA, introduced for the screening of at-risk subjects and to support diagnosis. However, ELISA tests used to detect anti-gliadin antibodies were not very specific; thus, other more sensitive and specific serological tests were later developed [6,33]. In particular, a test that showed a higher specificity as an anti-gliadin test, especially in IgA-deficient patients [34], was the test used to search for antibodies against deamidated gliadin peptides (DGP), which are modified by the TG2 enzymatic reaction called deamidation. Currently, tests based on the search for anti-DGP antibodies, together with those used for anti-TG2 antibodies (discussed later) are the most frequently employed serologic tests in clinical CD management. An alternative approach to detecting the autoimmune component is the use of IFAs. In these assays, the presence of autoantibodies in sera causes a specific staining pattern on tissue slides. Searching for anti-reticulin antibodies by IFA was an early method of screening. The term reticulin referred to a reticular component of connective tissue around smooth muscle fibres. A specific reticular pattern obtained by the staining of different rat tissues was considered to be associated with CD. Later, a more sensitive IFA test was employed to screen for the presence of anti-endomysial antibodies (EMA) [35]; endomysium is also a layer of connective tissue around smooth muscle cells, and EMA produce a characteristic staining pattern on slices of monkey oesophagus or of human umbilical cord. With the discovery that TG2 is the main CD autoantigen, it has been clarified that IFA staining patterns were due to the presence of TG2 in tissue slides.

### 2.8. CD Therapy

The only currently available therapy for CD is a strict lifelong diet without gluten [6]. Such a diet results in the remission of symptoms in about six months, accompanied by the recovery of intestinal mucosal architecture and the disappearance of the autoimmune antibodies. A gluten-free diet is often difficult to manage, and not only for social reasons. Indeed, gluten-free products and medicines often contain trace amounts of gluten as a consequence of cross-contamination during industrial production. Moreover, gluten-free products are poor in fibre, vitamins, and other micronutrients. Thus, the gluten-free diet tends to be unbalanced and requires additional nutrient supplementation [36]. A minority of adult CD patients are considered to be affected by “refractory CD”, when the disease does not respond to treatment with a gluten-free diet. Symptoms and intestinal atrophy persist even after 12 months of therapy [37]. These patients are at risk of developing serious complications, such as intestinal lymphoma and collagenous sprue. On the bases of increasing insights into CD pathogenesis, novel therapeutic strategies are under investigation, mainly intended as adjuvants to a gluten-free diet, as a rescue treatment after accidental or intentional gluten intake, or to manage refractory CD. One of these new approaches consists in the detoxification of gluten sequences using exogenous endopeptidases. Another possibility is gluten sequestration with polymeric binders, thus preventing immunogenic modification by TG2. Other strategies aim to interfere with pathogenicity patterns, for example by reducing intestinal permeability, by inhibiting the binding of gluten peptides to HLA molecules, by blocking proinflammatory cytokines, etc. Currently, several clinical trials based on above-mentioned strategies are under development, with encouraging results [38].

## 3. TG2

### 3.1. TG Family and TG Canonical Enzymatic Reactions

**Figure 2:**
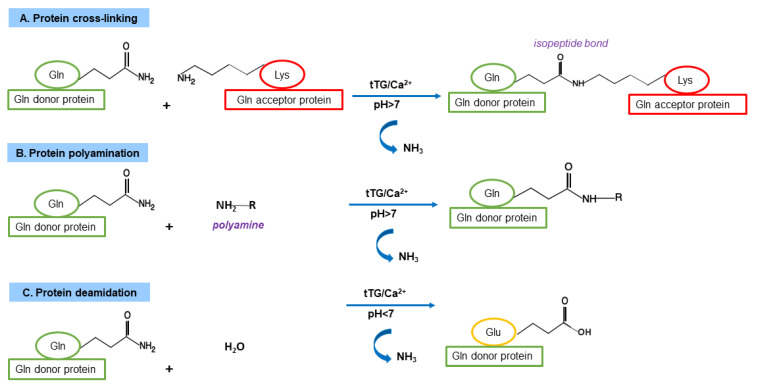
TG-catalysed acyl transfer reactions. (**A**). Cross-link formation between the γ-carboxamide group of a Gln residue and the ε-amino group of a Lys residue with the release of ammonia. (**B**). Reaction of incorporation of a polyamine into a Gln residue. (**C**). Deamidation of a Gln residue to a Glu residue.

The TG family includes transferase enzymes (EC 2.3.2.13), which catalyse the formation of an isopeptide bond involving the *γ*-carboxamide group of a Gln residue (the acyl-donor) and the *ε*-amino group of a Lys residue (the nucleophilic acyl-acceptor), generating an intra- or an inter-protein cross-link (Figure 2) [1]. The biochemical mechanism of the reaction involves a ping-pong kinetics: first, the *γ*-carboxamide group forms a thiol ester with the Cys residue of the enzyme active site with the release of ammonia, then the acyl intermediate is transferred to the nucleophilic aminic substrate. As alternatives to Lys residues as acyl-acceptors in the crosslinking reaction, TGs can use several low-molecular-mass primary amines (spermine, spermidine, putrescine, etc.). In the absence of suitable amines and in the presence of a slightly acidic pH, water can act as nucleophile; thus, TGs are able to deamidate Gln to Glu residues [1]. In mammals, nine structurally and functionally related genes encoding TGs have been identified [39]. Their main features are summarized in Table 1 [1,40]. Among them, only one gene encodes for a protein (band 4.2) without catalytic activity. All other eight mammalian TGs are strictly Ca^2+^-dependent enzymes and share a high sequence identity around the Cys proteinase-active site, comprising the catalytic triad Cys, His, and Asp [1]. Moreover, the structural organisation in four sequential domains is highly conserved in TG isoforms: the N-terminal *β*-sandwich domain is followed by the catalytic core domain (containing the Ca^2+^ binding sites) and two C-terminal *β*-barrel domains [1].

### 3.2. TG2 Expression, Localisation and Enzymatic Activities

In contrast to other TGs, TG2 has a wide distribution in tissues and cell types [1]. For this reason, it has been also named “tissue” TG. Its expression is constitutively high only in some localisations; for example, in the endothelium. However, TG2 transcription is upregulated by several activators; in TG2 promoter, response elements for transforming growth factor *β*1 (TGF-*β*1), IL6, nuclear factor k-B (NFkB), and retinoids have been found. Thus, TG2 expression appears increased as a consequence of tissue exposure to oxidative stress, O_2_ deprivation, and inflammatory stimuli [40,41].

The subcellular distribution of TG2 also renders it unique in the family of TGs [42,43]. Indeed, TG2 is mainly a cytosolic enzyme, but it migrates within the nucleus in variable amounts, depending on the signalling stimuli. TG2 is associated with the cell membrane surface, where it arrives by an unconventional secretion pathway in association with integrins [44], and is partially released into the extracellular matrix (ECM); it is also associated with the inner side of the plasmatic membrane, and is present in mitochondria in small amounts. Finally, it can be associated with the vesicular endosomal and autophagic compartments. Besides the typical enzymatic activities of the family (transamidation, deamidation, polyamination), in some conditions and localisations, TG2 displays other catalytic properties, i.e., GTPase, kinase, disulphide-isomerase, and isopeptidase activities. Moreover, some TG2 functions are not related to a specific catalytic activity, but are due to its non-covalent association with other cellular proteins [43].

### 3.3. TG2 Functions as Cross-Linking Enzyme

Canonical TG2 activities are strictly regulated by the availability of Ca^2+^ ions, and are thus virtually absent in cytosol, where Ca^2+^ concentration is very low. Each perturbation of Ca^2+^ homeostasis that mobilises Ca^2+^ from intracellular stores or from the extracellular environment could rapidly allosterically activate TG2. When Ca^2+^ binds TG2, enzyme domains move each other and protein assumes an “open” active conformation.

In the absence of Ca^2+^, and in the presence of GTP, a TG2 negative regulator, domains appear more compact, and the enzyme presents a “closed” inactive conformation [45,46]. At the extracellular level, Ca^2+^ concentration is enough to activate TG2, but the complete activation needs an appropriate redox state of the protein (reduced), which can be modulated by thioredoxin-1 and the endoplasmic reticulum (ER)-resident protein p57 [47,48]. TG2 transamidating activity is involved in several biological processes associated with the stabilisation of protein networks or modulation of functions of specific substrates, inside or outside the cell [49]. For example, in ECM and basal lamina, TG2 catalyses cross-links between proteins, such as collagen, fibrinogen, fibronectin, laminin and nidogen, giving strength and stability to ECM and also modulating cell-matrix adhesion [40,43]. In the cytosol, TG2 may modulate inflammation; one possible way is by forming polymers of IkB*α*, the inhibitor of NFkB; thus, NFkB becomes free to migrate into the nucleus to exert its transcriptional activity. In addition, TG2 may form cross-links in the anti-inflammatory peroxisome proliferator-activated receptor (PPAR)-*γ*, which is consequently degraded in proteasomes [40]. Moreover, TG2 has a complex role in modulating apoptosis [50]; depending on the context and type of stimulus, TG2 may have a pro-apoptotic role, for example in the modification of retinoblastoma, with consequent induction of the apoptotic program. In the late stage of apoptosis as well as during necrosis, the high Ca^2+^ level strongly activates TG2, leading to the stabilisation of a network of cellular proteins in dying cells before their clearance, thus preventing leaking of potential inflammatory components. TG2 transamidating activity also seems to have a strong role in autophagic flux, stabilising protein aggregates to target the autophagic vesicles [51]. In nuclei, TG2 may regulate gene expression by transamidating histones and other regulatory proteins, such as SP1 [40]. Finally, TG2-mediated addition of primary amines to several cytosolic substrates may modulate cytoskeleton rearrangements and vesicular trafficking [52].

### 3.4. TG2 Functions as Non-Crosslinking Enzyme

An intriguing property of TG2 is that it is a multifunctional protein displaying several catalytic activities and related biological functions, as well as catalytic-independent scaffolding/adapter/signalling functions [40,43]. Through its GTPase activity, TG2 acts as a non-canonical G protein at the level of the inner side of the plasmatic membrane. Associated with some G-protein coupled receptors, such as adrenergic receptors, oxytocin, and thromboxane A2 receptors, TG2 may participate in transducing signals by activating phospholipase C-*δ*1 [43]. GTPase activity is allosterically inhibited by Ca^2+^ ions, thus transamidating and GTP-hydrolysing activities are reciprocally regulated. A regulatory protein, calreticulin, binds to TG2 and inhibits GTPase function. Calreticulin dissociates from TG2 when the activated receptor causes TG2 to exchange GDP with GTP. Through its disulphide-isomerase activity, which is independent from both Ca^2+^ and GTP, TG2 may regulate mitochondrial physiology, in particular by acting on key components of the respiratory chain [43,53]. Histones, p53, and retinoblastoma proteins are possible substrates of the ATP-dependent kinase activity of TG2; through this activity, inhibited by a high level of Ca^2+^ ions, TG2 may regulate transcription and chromatin accessibility [40]. Finally, an isopeptidase activity of TG2 has been reported, but its possible physiological role is still uncertain.

On the cell membrane surface, TG2 may represent a co-receptor of fibronectin together with *β*1 and *β*3 integrin. Ternary complexes are formed in which TG2 collaborates to promote an out–in signal transduction in a catalytic-independent manner. TG2 also interacts with the extracellular domains of some growth-factor receptors (low-density lipoprotein receptor-related protein 1 (LRP1), platelet-derived growth factor) and some proteoglycans (syndecan-4), thus collaborating with their signalling functions [43]. Experimental evidence also indicates the occurrence of non-enzymatic functions of TG2 intracellularly; for example, TG2 may behave as a member of the BH-3-only subfamily of pro-apoptotic proteins. In addition, TG2 may display scaffolding properties, for example in association with AKAP13 and 14-3-3, through which it participates in several signalling pathways.

## 4. TG2 in CD Pathogenesis

Given the wide range of TG2 enzymatic and non-enzymatic functions and its ubiquitous distribution in tissues and cellular compartments, it is not surprising that TG2 has a role in the development of several human pathological conditions, such as cancer, neurodegenerative disorders, inflammatory and cardiovascular diseases, and autoimmune conditions [54,55,56].

Regarding CD, TG2 represents the trade union between the main environmental trigger (gluten) and the main genetic component (HLA genes) of CD. This enzyme, whose expression is greatly increased in inflamed intestinal sites both intracellularly (in enterocytes) and extracellularly (in the lamina propria) [57], recognises gluten fragments as preferred substrates and catalyses two crucial reactions: deamidation and transamidation of specific Gln residues.

### 4.1. Gluten Modification: Deamidation

**Figure 3:**
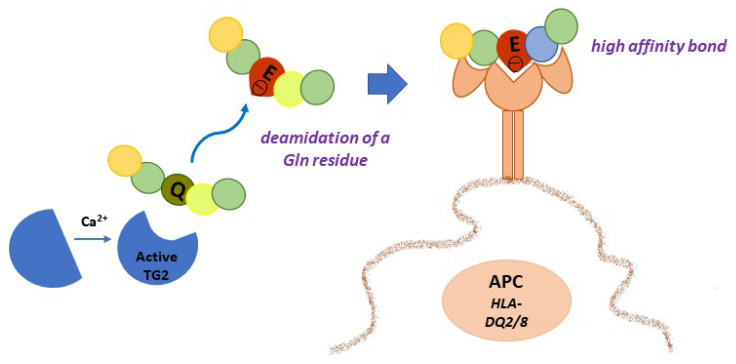
TG2 deamidation of a specific Gln residue in gliadin peptide introduces a net negative charge responsible for better recognition by DQ2/8 molecules on APC cells.

Given the high content of Gln residues in gliadin and other gluten proteins, it is evident that TG2 can use them as Gln-donor substrates in transamidating and deamidating reactions. Deamidation is not a common reaction because it requires specific conditions: a slightly acidic pH and a very low concentration of available amines. When and where these conditions may be realised in cells and tissues are still debated. In a recent work, it has been suggested that TG2 released in gut lumen as a consequence of enterocytes shedding could meet luminal gliadin, and in this way could have direct access to adaptive immune B cells at the level of Peyer’s patches [58]. TG2 on the cell surfaces of APCs could deamidate gliadin during its turnover by endocytosis at the level of acidic endocytic vesicles. The recent demonstration that TG2 is more abundant on the cell surface and in early endocytic vesicles of CD skin-derived fibroblasts than of control cells supports this hypothesis [27]. In any case, there is no doubt that TG2 recognises specific Gln residues in gliadin sequences and deamidates them, introducing a net negative charge that greatly increases the affinity with which DQ2/DQ8 heterodimers bind gliadin peptides for presentation to T cells [59] (Figure 3). Indeed, the production of high-resolution X-ray crystal structures of representative deamidated gluten peptides in complex with DQ2 and DQ8 has definitively confirmed the importance of the introduction of a negative charge at a specific location in a gluten peptide interacting with the HLA binding groove [60]. As a consequence, a stronger immune response is evoked. An updated list of CD-relevant deamidated gluten epitopes recognised by CD4^+^ T cells has recently been reported [10]. The majority of listed epitopes are DQ2.5-restricted and mainly belong to the α- and γ-gliadins, but some epitopes also belong to the ϖ-gliadins, glutenins, hordeins, secalins, and avenins. Only a few epitopes are DQ2.2-or DQ8-restricted and also belong to the α- and γ-gliadins and glutenins. This discrepancy is likely due to the fact that most gluten epitopes have been discovered in CD patients positive for HLA-DQ2.5 [10]. Thus, it is reasonable to expect that further additional epitopes will be defined in the future.

### 4.2. Gluten Modification: Mechanism of Anti-TG2 Antibody Production

As above reported, TG2 is the main autoantigen in CD. To date, no TG2-specific CD4^+^ helper T cells have been identified. For this reason, it is supposed that gliadin-specific CD4^+^ helper T cells are essential in the production of anti-TG2 antibodies at the intestinal level, as a consequence of a cross-linking reaction catalysed by TG2 and involving some gliadin peptides [2]. In this case, reactive Gln residues of gliadin peptides are acyl-donor substrates in the transamidating reaction. The aminic second substrates could be represented by Lys residues of TG2 itself or of other intracellular or surface-membrane proteins. Several models supported by experimental data have tried to explain how this activity could be responsible for the autoimmune response.

**Figure 4:**
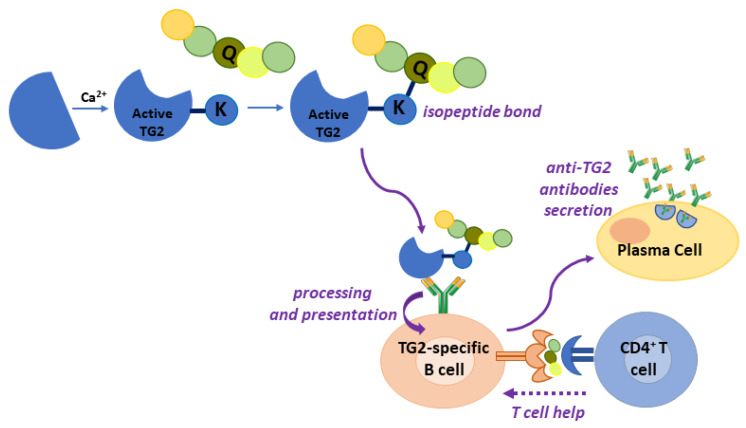
Gliadin peptides deriving from covalent complexes between TG2 and gliadin are presented by TG2-specific B cells, thus activating gliadin-restricted CD4^+^ T helper cells, which, in turn, stimulate plasma cell maturation and autoimmune response against TG2.

According to the classic hapten-carrier-like model (Figure 4), TG2 forms isopeptide bonds between itself (involving an autoreactive Lys residue) and gliadin peptides, generating complexes that are internalised, processed, and exposed by HLA-DQ2/8 molecules of TG2-specific B cells [61]. Then, these cells activate gluten-specific CD4^+^ T cells, which in turn stimulate B cells to produce anti-TG2 antibodies. In this regard, several isopeptides between TG2 and gliadin fragments have been identified [62]. Recently, du Pré et al. developed an elegant transgenic murine model demonstrating that autoreactive anti-TG2 B cells are able to produce autoantibodies when they receive help from T cells [63]. It has also been proposed that complexes between TG2 and gliadin could also be formed involving the Cys residue of the TG2 active site, with the formation of a thioester bond [64]. In any case, the hapten-carrier-like model better explains the occurrence of other autoantibodies besides anti-TG2. Indeed, other autoantigens have been identified (actin, desmin, calreticulin, collagen, etc.) and all can be recognised by TG2 as substrates for forming covalent complexes that could be processed by substrate-specific B-cells [65]. Moreover, this model is compatible with the observation that the autoimmune response gradually decreases when a gluten-free diet is adopted.

A revised version of the model proposes that TG2 is able to cross-link gliadin peptides directly to B cell receptors on TG2-specific B lymphocytes [66]. In a recent work [67], it has been demonstrated that the majority of TG2-specific B cells displayed epitope specificity for TG2 N-terminal sequences, and that cell-surface antibodies with such a specificity allowed TG2-cross-linking activity, leading to the formation of covalent complexes with gliadin and consequential deamidation during antigen presentation.

Another model on the generation of TG2 antibodies proposes that neoepitopes formed by the transamidating activity of TG2 targeting gluten peptides and itself could recruit T lymphocytes that escape thymic negative selection [2]. Finally, some evidence supports the idea that the autoimmune response against TG2 could involve a mechanism of molecular mimicry regarding viral or bacterial antigens [68]. However, the hypothesis of the occurrence of molecular mimicry between the rotavirus protein Vp7 and TG2 as a trigger of autoimmunity is currently uncertain [69].

### 4.3. Other TGs in CD

As mentioned above, autoimmunity in CD is attributed not only to TG2 but also to other TG family members. In particular, antibodies have been found against the human isoenzymes TG3 and type 6 TG (TG6). Furthermore, antibodies have also been detected against TGm, whose origin in the organism is a still debated issue.

#### 4.3.1. TG3 and TG6

TG3 and TG6 expression is mainly tissue-specific, and the appearance of antibodies targeting these isoenzymes is related to specific CD extraintestinal manifestations. TG3 is a zymogen that becomes active after a proteolytic cleavage, which gives a N-terminal subunit carrying the transamidating activity, and a C-terminal subunit [70]. TG3 is mainly expressed in hair follicles and epidermis, but it has been detected also in the brain, and in small amounts in the stomach, intestine, and testes. Its function is well-described only with regard to the epidermis, where it participates in the formation of the stratified squamous epithelium [71]; for this reason, TG3 is also called epidermal TG. Anti-TG3 antibodies are commonly present in the sera of patients affected by dermatitis herpetiformis (DH), which is a cutaneous disease characterised by blistering skin and intensive itching [72]. DH is probably the most common and specific extraintestinal manifestation of CD, occurring in about 25% of CD patients.

TG6 has been detected in testis and lung; it has also been studied in mouse brains where it may participate in neurogenesis and in the function of motor-neurons [73]. For this reason, TG6 is also called neuronal TG. A rodent model also highlighted a possible role of TG6 activity in the pathogenesis of Huntington’s disease [74]. The presence of circulating anti-TG6 antibodies and of their neuronal deposits is related to the neurological spectrum of CD. CD-associated neuropathy affects about 40% of CD patients and includes cerebellar ataxia (named gluten ataxia), cognitive impairment, headache, and neuropsychiatric disorders [75]. Unexpectedly, anti-TG6 antibodies are also very common in DH patients [76].

To date, the pathogenic role of TG3 and TG6 in CD-associated manifestations remains uncertain. However, both TG3 and TG6 are able to deamidate gluten peptides, albeit with a slightly different specificity with respect to TG2 [77]. They can also form covalent complexes with gluten peptides in vitro, even if with a lower efficiency; in particular, TG6, as with TG2, is able to form isopeptide and thioester bonds, whereas TG3 is able to form only thioester bonds [77]. Thus, it can be speculated that other TG isoenzymes besides TG2 may form complexes with gluten and generate autoantibodies according to the hapten-carrier-like model. Recent evidence indicates that DH patients possess plasma cells secreting anti-TG3 antibodies (and probably also anti-TG6 antibodies) in lamina propria [78], indicating that, as with anti-TG2 antibodies, antibodies targeting other TG isoenzymes are also generated at the gut level.

#### 4.3.2. TGm

Addressing TGm in the context of CD involves the investigation of two kinds of enzyme. The first is the exogenous TGm, mainly deriving from the microorganism *Streptomyces mobaraensis*, which is widely employed as a crosslinking agent in food industry [79]. TGm from *S. mobaraensis* is a Ca^2+^-independent enzyme able to transamidate a variety of different substrates in a wide range of pH levels and temperatures. Thus, it is an ideal enzyme to improve texture, stability, elasticity, nutritional content, and other qualities of several food types, including meat, fish, bakery, and milk products. TGm is an additive in processed foods, and as a consequence, it is introduced in variable doses into the organism through the diet. The second kind of TGm is an enzyme that may be produced and released endogenously by intestinal microbiota, or even occasionally by pathogens (bacteria and fungi) [80]. In a recent work, it was demonstrated that commercial TGm can be transported into enterocytes, where it is localised at the apical membrane, at the basolateral membrane, and in ER, and can also colocalise with endocytosed gliadin. Consequently, gliadin deamidation by TGm and activation of the immune system can be hypothesised [81]. Moreover, it has been suggested that TGm is immunogenic in CD patients, as circulating antibodies (in particular of IgG class) against TGm have been detected in a paediatric population. TGm immunogenicity appeared enhanced when TGm formed covalent complexes with gluten peptides, generating neoepitopes [82]. These observations led some authors to propose the antibodies targeting these neoepitopes as a new marker for CD screening, diagnosis, and predictability [83].

### 4.4. Potential Pathogenetic Role of Anti-TG2 Antibodies

A significant correlation between TG2 antibody titres and the degree of damage at the level of intestinal mucosa has been reported [84]. However, there is no clear evidence indicating a specific pathogenetic role for TG2 autoantibodies. A number of studies, sometimes with discordant results, have tried to clarify the biological effects of these antibodies, suggesting that their presence in the intestine and other areas could be a contributing factor in disease development [85,86]. Here, we briefly focus attention on the features of CD mucosal lesion that could be induced by anti-TG2 antibodies. In in vitro models, anti-TG2 antibodies, by interacting with membrane surface TG2, are able to promote cell proliferation and reduce differentiation [87,88]; they also cause actin cytoskeleton reorganisation, altered permeability, and phosphoproteomic changes [87,89,90]; and they mobilise Ca^2+^ ions from intracellular stores causing consequential TG2 activation [91]. Moreover, they seem to disturb some steps of angiogenesis [92]. In a murine model, the intraperitoneal injection of CD anti-TG2 antibodies caused the appearance of an altered intestinal mucosal morphology, even if clinical features of CD were not evident [93]. Interestingly, several described biological effects of anti-TG2 antibodies are the same as those evoked by P31–43, such as actin rearrangement, S-phase entry, and Ca^2+^ mobilisation from intracellular stores (Table 2) [57]. These observations suggest that anti-TG2 antibodies could act, at least in part, on the same pathways engaged by P31–43, with unknown potential synergic effects.

Circulating IgA and IgG anti-TG2 may also reach several tissues and organs. Deposits of anti-TG2 antibodies have been found in the liver, kidney, lymph nodes, and muscles of CD patients, where they could contribute to some extra-intestinal manifestations of CD [21,85]. Finally, the presence of anti-TG3 and anti-TG6 antibodies led to the hypothesis that these antibodies also could have a role in CD skin and neurological manifestations. In this regard, the intraventricular injection of anti-TG2/TG3/TG6 cross-reactive autoantibodies induces ataxia in mice [94]. Moreover, anti-TG3 antibodies are able to induce DH-like pathology in human-skin-grafted SCID mice [95].

Interestingly, an idiotypic humoral response against anti-TG2 antibodies has recently been detected in CD patients without active mucosal lesions, but was undetectable in patients with active lesions, leading to the hypothesis that the idiotypic response could serve to counteract the potential negative effects of the idiotypic response [96].

**Table 2:** Biological effects evoked by both anti-TG2 antibodies and P31–43 in human in vitro models.

Observed Biological Effect	Study Model for Anti-TG2 Antibodies	Study Model for P31–43
increased proliferation	Caco-2 cells [97]; enterocyte from CD patients on gluten-free diet [87]	Caco-2 cells [97]; enterocyte from CD patients on gluten-free diet [98]
reduced epithelial growth factor (EGF) endocytosis	Caco-2 cells [97]	Caco-2 cells [98]
increased ERK phosphorylation	Caco-2 cells [97]	Caco-2 cells [98]
actin rearrangement	Caco-2 cells [97]	Caco-2 cells [98]; enterocyte from CD patients on gluten-free diet [98]
Ca^2+^ mobilisation from ER and mitochondria	Caco-2 cells [91]	Caco-2 cells [99]
intracellular TG2 activation	Caco-2 cells [91]	Caco-2 cells [99]

### 4.5. TG2 and Gliadin Handling by Cells

TG2 seems to have a role in modulating gliadin peptide handling. This observation comes mainly from evidence regarding the effect of anti-TG2 antibodies on peptide uptake or transepithelial passage. For example, a study demonstrated that antibodies to TG2 enhanced the transepithelial passage of peptide 57–68 and P31–43 across a layer of Caco-2 cells [100]. With respect to peptide endocytosis, it has been reported that anti-TG2 antibodies specifically deranged P31–43 uptake (and reduced its consequential biological effects), but not peptide 57–68 uptake by Caco-2 cells [97]. The specificity of the action of anti-TG2 antibodies towards P31–43 led to the hypothesis that a receptor was involved and that this receptor could be TG2 itself. However, the attempt to isolate a receptor/carrier for P31–43 did not give positive results [101]; on the contrary, experimental data suggested that P31–43 could enter into the cell by a direct interaction with the cell membrane, and that it could be the prototype of a new class of cell-penetrating peptides. Thus, it can be supposed that anti-TG2 antibodies, by modulating endocytosis of TG2 and its partners, also modulate the recycling of near membranes, which may be interacting with P31–43.

### 4.6. TG2 Contribution to Coeliac Cellular Phenotype

Findings that anti-TG2 antibodies derange P31–43 uptake by Caco-2 cells led to the supposition that these antibodies could have a protective role against the negative effects of such a peptide. However, this hypothetical protective role seemed to fail in CD skin-derived fibroblasts, while it was observed in the control skin-derived fibroblasts [102]. The reason for such different behaviour in CD cells is still uncertain, but could be in some way related to TG2 properties. For example, TG2 appears differently distributed in CD and control fibroblasts; it is more associated with cell membranes in CD cells, in particular with cell surface and early endosomal and autophagic compartments [27]. Furthermore, P31–43 induces TG2 expression in CD cells, but not in control cells, whereas the same peptide activates more TG2 intracellular activity in control cells than in CD cells [27]. Thus, TG2 and anti-TG2 antibodies may contribute in some way to the definition of the coeliac cellular phenotype.

It is important to underline that currently, there is no experimental evidence on a possible contribution to CD pathogenesis by the non-canonical enzymatic activities of TG2. Given the peculiar TG2 localisation and activation in CD cells, it is reasonable to suppose that other TG2 functions could be implied in defining the coeliac cellular phenotype.

## 5. TG2 in CD Diagnosis

### 5.1. EMA and ELISA Tests for TG2

In 1997, the main autoantigen of anti-reticulin antibodies and EMA was identified as TG2 [20], and this discovery opened the way to the development of ELISA to detect TG2 in sera. The first ELISA worked using guinea pig TG2 as an immobilised antigen in test plates. Then, recombinant human TG2 became the elective antigen in these tests, which gradually reached high sensitivity and specificity. Tests based on TG2 antibody detection appeared to be slightly more sensitive compared to those based on EMA detection, but less specific [21]. On the other hand, IFA methods were more laborious, involved ethical concerns (with respect to the use of monkey oesophagus), and were to some degree operator-dependent. Thus, due to their accuracy and their non-invasive nature, ELISA tests for the detection of anti-TG2 antibodies currently remain the most commonly used test in the CD diagnostic workup. In a recent work, several synthetic complexes between TG2 and gluten peptides were generated and tested for their immunogenicity. Different neoepitopes were identified that were also recognised by the sera of CD patients on gluten-free diets with various degrees of mucosal healing [34]. Authors proposed these new biomarkers not only for diagnosis but also for monitoring the mucosal healing process. Fingertip-based tests searching for anti-TG2 or anti-DPG antibodies in whole blood are also available and offer the advantage of being a rapid method of initial screening for CD, along with the benefits of cost reduction and rapid response [33,103]. The presence of serum anti-TG2 antibodies, together with EMA positivity, is very often a hallmark of a subtype of CD, defined as “potential CD”, including cases with predisposing genes HLA-DQ2/DQ8, with middling or no symptoms, and without villous atrophy, representing about 10% of total CD cases.

Recent guidelines for diagnosis from the European Society for Paediatric Gastroenterology Hepatology and Nutrition (ESPGHAN) [104] substantially confirmed previously released guidelines (2012) indicating that, in children with suspected CD, duodenal biopsies are not necessary for the diagnosis in cases where anti-TG2 antibodies titres are more than 10 times higher than the upper normal limit. In these guidelines it is also recommended to test total IgA and anti-TG2 IgA as an initial screening in clinical practice for children with suspected CD, while tests searching for EMA, anti-DGP, or anti-gliadin antibodies are not recommended in initial screening. In patients affected by selective IgA deficiency, a 10–20-fold increased risk of CD development may be observed. Thus, in these subjects, screening for CD is mandatory. Such a screening is effectively based on the detection of circulating IgG anti-TG2 antibodies [6].

### 5.2. Detection of Intestinal Anti-TG2 Antibodies

Since anti-TG2 antibodies are produced at the intestinal mucosa level and only later spill off in blood vessels, methods to detect them in situ could have a diagnostic utility in situations of doubt (for example, in patients with negative serology) or when other approaches are inconclusive [105,106]. First detection of intestinal anti-TG2 antibodies is performed on faecal supernatants. Alternatively, antibodies produced by cultured intestinal biopsies can be detected by both IFA and ELISA tests, or can be detected by immunofluorescence as IgA deposits in a section of duodenal mucosa. These deposits seem to disappear very slowly from the intestine, where anti-TG2 antibodies are still produced for a long time after the introduction of a gluten-free diet, even if they are no longer detectable in serum [107]. It has been proposed that the detection of mucosal deposits of anti-TG2 antibodies could have a predictive value with respect to the successive development of villous atrophy in potential CD patients. Moreover, anti-TG2 IgA deposits have been also detected in at-risk subjects without antibodies in serum, thus possibly indicating a very early stage of the disease. However, in these cases it is not possible to exclude that the presence of anti-TG2 antibodies at mucosal level may not be specific for CD, but due instead to an inflammatory state related to another autoimmune condition, as in the case of patients affected by type 1 diabetes [105]. Finally, in patients with selective IgA deficiency, a condition frequently associated with CD, compensatory IgM anti-TG2 antibody deposits have been found at the gut level [108].

### 5.3. Anti-TG3 and Anti-TG6 Antibodies in Clinical Practice

The serology of DH patients is very similar to that of CD patients presenting classical intestinal symptoms. However, in addition to intestinal and circulating anti-TG2 antibodies, anti-TG3 antibodies can be detected in the serum of about 90% of DH patients, whereas IgA-TG3 deposits are present in the papillary dermis of 100% of DH patients [23]. The autoimmune response to TG3 is strictly gluten-dependent, as antibodies gradually disappear during a gluten-free diet, together with skin symptoms. The diagnosis of DH is based on the IFA detection of IgA anti-TG3 deposits complexed with TG3 in lesional biopsies of dermal papillae and/or dermoepidermal junctions. However, serological tests aimed at detecting circulating IgA anti-TG3 are used to further aid diagnosis [109].

Circulating anti-TG6 (besides anti-TG2) are detected in the sera of patients affected by neurological manifestations, and form deposits in neuronal tissues; as for anti-TG3 antibodies, their presence is strictly gluten-related and the anti-TG6 titre decreases in parallel with the gluten-free diet. Serological tests aimed at detecting anti-TG6 antibodies are considered specific and sensitive tools for diagnosis of CD-related gluten ataxia and other neurological complications [110].

On the whole, even if the diagnostical helpfulness of circulating anti-TG3 and anti-TG6 antibodies is well assessed, it has been considered that the absence of autoantibodies does not preclude the presence of antibodies in the target organs, i.e., skin and brain, respectively, as also shown for anti-TG2 deposits.

## 6. TG2 in CD Therapy

Increased TG2 expression in CD intestinal lesions is a consequence of inflammatory stimuli, including gliadin peptides [57]. On the other hand, TG2 deamidation potentiates gluten antigenicity, thus enhancing T cell activation and inflammation, inducing a vicious circle that leads to further TG2 upregulation [2]. Moreover, TG2 provokes the release or activation of inflammatory mediators, such as TGF-*β*1, NFkB, and phospholipase A2. Therefore, TG2 is a promising target for therapeutical strategies aimed at blocking its activity, with the double aim of reducing deamidation and controlling inflammation. However, TG2 also has a role in repairing damaged mucosa, as it contributes to ECM remodelling. Thus, therapeutical strategies based on TG2 inhibition must take account of this beneficial role of TG2. The development of in vitro and in vivo models consisting in enterocyte cell lines, primary intestinal and non-intestinal cells, co-culture with cells derived from the immune system, and transgenic mice expressing human HLA-DQ2/8 genes, has favoured studies investigating the causes and consequences of TG2 activation in the context of CD [60]. In these models, the use of TG2 inhibitors have helped to confirm and to better define the role of TG2 in CD pathogenesis and have opened the way to experimentation with some drugs in preclinical and clinical trials with encouraging results.

### 6.1. Studies on In Vitro TG2 Inhibition

The first inhibitors tested to investigate the role of TG2 in some pathological conditions were primary amines and other aminic pseudo-substrates, which, acting as competitive aminic substrates, prevented the formation of naturally isopeptide bonds; they were effective at very high concentrations in a non-specific way, and thus their therapeutical potential appeared very limited; however, they were useful to identify several Gln-donor TG2 substrates [111]. Another group of reversible inhibitors contained non-hydrolysable GTP analogues able to stabilise the inactive closed TG2 conformation [54]. Additionally, competitive peptidic substrates, both synthetic (for example proelafin fragment) and natural (for example, trigedin), were tested, and some of them demonstrated good inhibitory ability [111].

In parallel, several site-direct irreversible inhibitors were discovered or developed. Among the first chemical inhibitors tested in CD models was cystamine, an irreversible thiol-reactive reagent that blocks the active site of the enzyme. When cystamine was added at high concentrations to the intestinal biopsies of CD patients, it inhibited the proliferation of gluten-specific T cells [112]. However, it was not specific for TG2, and other Cys-enzymes could be affected. More selective irreversible inhibitors were also developed; given their higher affinity for TG2, they were more suitable for potential therapeutical usage. Derivatives of non-peptidyl 2-[(2-oxopropyl)thio] imidazolium (the membrane-permeable R283 and the membrane-impermeable R281) were employed to demonstrate the role of TG2 in mediating some gliadin effects. For example, in Caco-2 cells, they displayed a protective effect towards modifications induced by peptic-tryptic digested gliadin on transepithelial resistance, actin rearrangement, and junction protein expression [113]. Another class of compounds with a high specificity for TG2 was that of the 6-diazo-5-oxo-norleucine (DON) derivatives. DON is a glutamine antagonist able to inhibit several Gln-utilising enzymes through an irreversible alkylation. DON compounds are short peptides derived from gluten sequences in which a Gln residue is replaced by a DON residue to generate ligands, which bind to the TG2 active site with nanomolar affinity. In a three-dimensional cell co-culture model, DON compounds showed the ability to inhibit enterocyte differentiation without cytotoxicity [114].

Finally, a series of 3-halo-,4-,5-dihydroisoxazole derivatives were developed from the structure of acivicin, a Gln analogue able to inhibit Cys-enzymes [115]. The first developed moieties showed cross-reactivity with other TG isoforms, and thus modifications were made to obtain compounds with higher potency and selectivity. Three of these improved compounds, CK996, ZH147A, and CK805, deriving from reference moiety ERW1041E, showed the best pharmacokinetic profiles, thus being of interest for future evaluation of their biological activity in the context of CD [116].

Interestingly, the recent development of peptidomimetic covalent inhibitors able not only to block transamidating activity but also to allosterically abolish the ability of TG2 to bind GTP [117] could open the way to future studies investigating the involvement of TG2 GTPase function in CD.

### 6.2. Studies on In Vivo TG2 Inhibition

To select an optimal TG2 inhibitor for in vivo studies in the CD context requires identification of a compound that reaches lamina propria and is present in the adequate concentration and for sufficient duration to inhibit the target, without cytotoxicity and without cross-reactivity with other locally present members of the TG family such as Factor XIIIa, which is indispensable for the coagulation cascade. Another consideration is that some gluten peptides may be immunogenic without TG2 deamidation, thus it is necessary to hypothesise that other drugs must be combined with a TG2 inhibitor to reduce the quantity of gluten peptides reaching lamina propria. Studies on TG2 inhibition in vivo have been conducted on mice models, and in some cases, they have been considered as preclinical experimentations.

The first evidence for TG2 inhibition in mammalian intestine was given by a study on a mouse model of intestinal inflammation [118]. Intraperitoneal injection of polyinosinic-polycytidylic acid induced an inflammatory response in the small intestine associated with an increased TG2 activity. The oral administration of the dihydroisoxazole inhibitor ERW1041E reduced TG2 activation, thus counteracting intestinal inflammation. Similar results were also obtained using a DON compound of the new generation, ZED1227 [119,120]. Furthermore, in a transgenic mouse model overexpressing IL15 at the intestinal level and expressing DQ8 molecules, a gluten-containing diet caused mucosal atrophy, increased TG2 expression and activity and humoral response against gliadin, and deamidated gliadin peptides [121]. Oral administration of dihydroisoxazole derivatives ERW1041E and CK805, in the presence of gluten, inhibited intestinal TG2 activity and prevented the production of anti-gliadin peptide antibodies and mucosal atrophy [121]. This work confirms the crucial role of TG2 in CD pathogenesis. In conclusion, currently available data indicate that ERW1041E, CK805, and ZED1227 may represent promising orally available drugs, being potentially able to block inflammation induced by TG2 deamidation.

### 6.3. Anti-TG2 Antibodies as Blocking Agents

Anti-TG2 antibodies have often been employed as modulators of TG2 functions, and their potential as a therapeutical tool has been explored for some time. However, antibodies do not penetrate into the cell, and thus may exert their action on membrane-bound TG2 or on the enzyme in the ECM. In a recent work, high-affinity TG2-targeted antibodies have been obtained by hybridoma technology; the ability of these antibodies to inhibit the activity of extracellular TG2 and consequently to block ECM accumulation in a cell model of fibrosis renders them suitable for future clinical development for fibrotic conditions [122]. In the context of CD, several studies have investigated the ability of antibodies from CD patients to display an inhibitory effect on transamidating activity of purified recombinant or commercial TG2 [85]. When tested on cell-surface TG2, antibodies inhibited transamidating activity in a very limited way, or even activated TG2 [85]. However, in some experimental models, anti-TG2 antibodies exerted a beneficial effect by counteracting P31–43 biological activity, for example in experiments where P31–43 induced proliferation or inflammation [57]. Given the ability of anti-TG2 antibodies to derange P31–43 uptake by some cells, it is reasonable to assume that their effect on P31–43 activity is due to a reduction in peptide entrance into the cells and not to a modulation of TG2 surface activity; indeed, TG2 competitive substrates or inhibitors had no effect on P31–43 internalisation [97].

### 6.4. TG2 Inhibition in Clinical Trials

The first TG2 inhibitor to have successfully arrived at the conclusion of phase I and phase II clinical trials for CD treatment is the dihydroisoxazole derivative ZED1227 [123], produced by Zedira pharmaceuticals. In the phase II randomised, double-blind, placebo-controlled trial, the efficacy and safety of ZED1227 capsules was established. The study was conducted on about 160 adult CD patients in histologic and serologic remission who were challenged with 3 g of gluten daily for 6 weeks. Patients were assigned to three groups, each treated with a different dose of ZED1227 (10, 50 and 100 mg), whereas a fourth group received a placebo. The primary endpoint of the study aimed to evaluate the reduction in gluten-induced damage in terms of ratio of villus height to crypt depth. Secondary endpoints were relative to changes in the density of intraepithelial lymphocytes and to patient-reported outcomes, including questionnaires for assessment of health-related quality of life, serological CD markers, and markers of malabsorption. Safety of ZED1227 administration was evaluated by monitoring parameters including adverse and side-effect events, vital signs, body weight, and other laboratory tests. Efficacy results were positive (i.e., attenuation of mucosal damage) in all three study groups with respect to the placebo group. Additionally, the increase in intraepithelial lymphocytes was dose-dependently attenuated by ZED1227. A positive response for other secondary end points was registered, particularly at the 100-mg dose. Common adverse effects in all groups (placebo group included) were headache, nausea, diarrhoea, vomiting, and abdominal pain. Although this phase II clinical trial clearly demonstrated that TG2 inhibition by ZED1227 attenuated gluten-induced mucosal deterioration, further studies in a higher number of patients are necessary to better characterise the efficacy and safety of ZED1227 treatments. However, the data encourage the planning of a confirmatory phase III trial.

Currently, another irreversible TG2 inhibitor is under preliminary clinical investigation. A phase I trial is underway with GSK3915393, a new compound produced by GlaxoSmithKline (ClinicalTrials.gov Identifier: NCT04604795).

## 7. Gluten Transamidation as a Detoxification Strategy for Therapeutical Approach

Enzymatic modification of gluten peptides is one of the approaches under investigation to reduce gluten immunogenicity and toxicity [38]. The use of bacterial prolyl endopeptidases can reduce long undigested gluten peptides to short peptides without immunogenic activity [124]. A different approach is to explore the possibility of modifying gluten sequences and reducing their immunogenicity while preserving the integrity of the protein structure. This approach consists of transamidating gluten sequences with a food-grade TG [125]. Initial attempts to reduce gluten immunogenicity were made by inducing the formation of cross-links between gluten sequences [126], but encouraging results were obtained when transamidation was performed in the presence of an adequate amine-donor, such as L-Lys, L-Lys methyl or ethyl esters, or n-butylamine, in attempts to reduce the affinity of modified gluten sequences binding to DQ2/8 molecules [127,128,129]. The use of the commercial guinea pig TG2 and of the recombinant human TG2 for gluten transamidation was initially explored [127,128]. In particular, TG2 treatment of peptic-tryptic digested gliadin from wheat flour in alkaline conditions (to avoid deamidation) in the presence of Lys methyl ester caused a significant reduction in CD-derived gliadin-specific T cell activation and INF*γ* production, indicating that transamidation abolished the immune reactivity of several gliadin epitopes [127]. Since TGm is already available as a food-grade enzyme in several industrial applications, TGm appears to be a good candidate for use in gluten transamidation. Effectively, TGm showed the same specificity as TG2 with respect to the Gln residue target of the enzymatic reaction [130], but TGm offered a great advantage in that it poorly deamidated gliadin, even though the reaction was conducted in water. Moreover, the treatment of the gliadin present in wheat flour with TGm and Lys methyl ester was effective in inhibiting T cell activation. In vivo experiments conducted on HLA-DQ8 transgenic mice demonstrated that treatment with transamidated gliadin produced a modification of the ratio IL10/INF*γ*, indicating the ability of transamidated gliadin to convert a mainly inflammatory phenotype to a mainly anti-inflammatory phenotype [131]. Similar results were obtained in human biopsy samples [4]. In addition, in a DQ2 mouse model, a transamidated purified *α*-gliadin fraction, and a single recombinant *α*-gliadin sequence were able to counteract the pro-inflammatory activity of native gliadin [4,132]. A possible objection to the enzymatic approaches here described is the consideration that, even if TG enzymatic transamidation targets Gln residues responsible of immunogenicity when deamidated, a number of other immunogenic epitopes that do not need deamidation to bind HLA-DQ2/8 molecules could remain unaltered. Moreover, TG modification may not be directed to the sequences responsible for innate immune response. Nevertheless, on the basis of scientific evidence, enzymatic therapy to reduce gluten immunogenicity by transamidation appears to be a possible therapeutical approach, at least with an adjuvant role, and is suitable for further in-depth analysis. In this context, the first clinical trials have been conducted with the primary aim of evaluating the ability of gluten-transamidation with Lys methyl ester to maintain CD patients (adhering to a gluten-free diet) in clinical remission and also to prevent the inflammatory response. The studies demonstrated that the administration of bread/rusks prepared with TGm-transamidated wheat flour ameliorated symptoms and led to a reduced number of relapses in challenged patients [133,134].

## 8. Conclusions

It is doubtless that TG2 represents a key enzyme in the pathogenic mechanisms leading to CD development. At the site of intestinal inflammation, where TG2 is typically overexpressed, its canonical enzymatic activity leads to the formation of isopeptide bonds between itself (or other self-proteins) and gluten peptides, thus provoking a strong autoimmune response. Still uncertain is the role of circulating and intestinal anti-TG2 antibodies in contributing to CD onset; however, antibody detection represents an irreplaceable tool for CD screening programs and diagnosis. In particular circumstances (low pH and virtual absence of primary amines) in the CD mucosa, TG2 can modify specific Gln residues of gluten peptides, transforming a neutral residue into an acidic residue (Glu), which is important in the presentation to the immune system by DQ2/8 molecules and the consequent loss of oral tolerance towards gluten. For these evident reasons, TG2 has become the target of recent pharmaceutical approaches aimed at reducing deamidation. New generations of increasingly specific and potent TG2 inhibitors are under investigation, some of which have reached the point of clinical experimentation. Finally, given the increasing use of food-grade TGs, particularly TGm, as biotechnological tools in a number of industrial applications, some researchers are exploring the possibility of modifying gluten peptides by transamidating them with nucleophilic amines in an attempt to reduce the immunogenicity of the majority of DQ2/8-restricted epitopes.

Finally, it is appropriate to underline that TG2 is a multifunctional enzyme with several catalytic activities and non-catalytic functions. Thus, it cannot be excluded that TG2 may have other unexpected roles in CD pathogenesis, independent of its transamidating and deamidating activity. For example, TG2 is easily targeted on the cell surface by anti-TG2 antibodies, and not only at the intestinal level. Given the biological consequences of such an interaction at the cell surface (for example, increased proliferation and structural and signalling modifications) and potential differences in subcellular TG2 distribution in CD cells, it is reasonable to conclude that TG2 may contribute to CD onset and progression in other peculiar and still unexplored fashions.

## Figures and Tables

**Table 1:** Mammalian TGs. For each isoenzyme, the gene name, protein name, main localisations, and main biological functions are listed.

Gene Name	Protein Name	Localisation	Biological Functions
TGM1	keratinocyte or type 1 TG (TG1)	keratinocytes (cytosol and plasma membrane)	cornified envelope formation
TGM2	tissue or type 2 TG (TG2)	ubiquitarian (cytosol, nucleus, membranes, mitochondria, ECM)	signalling, differentiation, apoptosis, ECM stabilisation, tissue repair
TGM3	epidermal or type 3 TG (TG3)	epidermal cells and hair follicles (cytosol)	cornified envelope formation
TGM4	prostate or type 4 TG (TG4)	prostate and prostatic fluids (secreted)	semen coagulation
TGM5	type 5 TG (TG5) or TGx	mainly in epithelial and skeletal muscle cells (cytosol)	cornified envelope formation
TGM6	type 6 TG (TG6) or TGy	nervous, lung and testis cells	nervous system development
TGM7	type 7 TG (TG7) or TGz	quite ubiquitarian, mainly in lung and testis cells	unknown
FXIIIA1	plasma TG or Factor XIIIa	mainly in macrophages and platelets (extracellular)	blood clotting, tissue repair
EPB42	band 4.2 (B4.2) or erythrocyte membrane protein B4.2	mainly in erythrocytes (surface membrane bound)	structural, in plasma membrane

## Data Availability

Not applicable.

## Abbreviations

APCs	antigen-presenting cells
CD	coeliac disease
DH	dermatitis herpetiformis
DPG	deamidated gliadin peptides
ECM	extracellular matrix
ELISA	enzyme-linked immunosorbent assay
EMA	anti-endomysial antibodies
ER	endoplasmic reticulum
HLA	human leukocyte antigen
IFA	indirect immunofluorescence assay
IL	interleukin
INFγ	interferon-γ
NFkB	nuclear factor k-B
P31–43	α-gliadin peptide 31–43
TG	transglutaminase
TG2	type 2 transglutaminase
TG3	type 3 transglutaminase
TG6	type 6 transglutaminase
TGF-β1	transforming growth factor β1
TGm	microbial transglutaminase
